# Nonsyndromic intellectual disability with novel heterozygous *SCN2A* mutation and epilepsy

**DOI:** 10.1038/s41439-018-0019-5

**Published:** 2018-07-20

**Authors:** Takayuki Yokoi, Yumi Enomoto, Yoshinori Tsurusaki, Takuya Naruto, Kenji Kurosawa

**Affiliations:** 1 Division of Medical Genetics, Kanagawa Children’s Medical Center, Yokohama, Japan; 2 Clinical Research Institute, Kanagawa Children’s Medical Center, Yokohama, Japan; 3 Pediatrics and Developmental Biology, Tokyo Medical and Dental University Graduate School, Tokyo, Japan

## Abstract

*SCN2A* mutations are primarily associated with a variety of epilepsy syndromes. Recently, *SCN2A* has been reported as a gene responsible for nonsyndromic intellectual disability or autism spectrum disorders. Here, we present a case of a 12-year-old girl with nonsyndromic intellectual disability who exhibited a heterozygous de novo missense mutation in *SCN2A*. She developed seizures during the course of illness. This case suggests that the phenotype of patients with heterozygous *SCN2A* mutations can be variable.

The sodium voltage-gated channel alpha subunit 2 (*SCN2A*) gene is mainly and highly expressed in the brain^1^. Many heterozygous variants of *SCN2A* are associated with a variety of human epilepsy syndromes^2,3^. Many are benign syndromes such as benign familial neonatal-infantile seizures (BFNIS), benign familial infantile seizures (BFIS), generalized epilepsy with febrile seizures plus (GEFS+), and intractable childhood epilepsy. Furthermore, *SCN2A* is occasionally responsible for Dravet syndrome, which is a rare and malignant epilepsy syndrome, usually developing in the first year of life^4^. Dravet syndrome is now used as a disease entity, which includes severe myoclonic epilepsy in infancy (SMEI) and its borderline phenotype (SMEB). The functional consequences of mutant sodium channels range from loss to gain of function. *SCN2A* mutations associated with intractable epilepsy alter the channel properties of the sodium channel, voltage-gated, type II (Nav1.2) to a greater extent than BFNIS mutations do, suggesting a mechanism for more severe epileptic phenotypes^5^. However, other phenotype–genotype correlations are unknown for *SCN2A* mutations. Moreover, *SCN2A* appears to be one of the major genes in which mutations are detected in nonsyndromic intellectual disability (ID) or autism spectrum disorders (ASDs) by whole-exome sequencing^6^. Herein, we present a case of an atypical patient with nonsyndromic ID and with a heterozygous missense mutation detected by clinical exome sequencing. Although the patient was assumed to have nonsyndromic ID only, she developed seizures during the course of her illness.

The patient was a 12-year-old Japanese girl born to nonconsanguineous healthy parents, with no family history of the disease. The pregnancy was uneventful. The girl was born at 39 weeks of gestation with a birth weight of 2868 g, length of 49 cm, and head circumference of 33 cm.

Her motor development milestones were within the normal limit. However, it was noted that she spoke no words and was not able to point to an object at one and a half years old, and consequently, she was referred to our hospital. Examinations including brain MRI did not determine the cause of her developmental delay. She was developing slowly, never showed any progressive symptoms, and was followed up as a case of nonsyndromic ID of unknown cause. She began to speak single meaningful words at the age of 5 years. Her height, weight, and head circumference were appropriate for her age. She could speak two-word sentences but often repeated words or phrases. Electroencephalogy (EEG) showed spike bursts in the bilateral parietal/occipital region since one and a half years old. However, she had no seizures, nor did she have any other symptoms, dysmorphic appearance, or complications. Her intelligence quotient was 36–50. Karyotyping and microarray comparative genomic hybridization found no pathogenic variants. She developed absence seizures and began taking valproic acid, which controlled the seizures, at the age of 12.

Written informed consent was obtained from the parents of the patient in accordance with the requirements of the Kanagawa Children’s Medical Center Review Board and Ethics Committee.

Total genomic DNA was obtained from lymphocytes using the QIAamp DNA blood mini kit (Qiagen, Valencia, CA, USA), following the manufacturer’s instructions.

DNA libraries were enriched for sequences using the TruSight One sequencing panel kit (Illumina, Inc., San Diego, CA, USA), which enables enrichment and final analysis of a panel of 4813 genes. Patient samples were sequenced using the MiSeq platform (Illumina, Inc.), with 150-bp pair-end reads. Data were analyzed by the Burrows-Wheeler alignment tool and the Genome Analysis Toolkit pipeline (Broad Institute, Cambridge, MA, USA) and visualized in the Integrative Genomics Viewer (IGV). Calling copy number variation (CNV) was based on log-ratio analysis and read depth (*z*-score) of each exon. Mutations identified by targeted sequencing were confirmed by Sanger sequencing, and appropriate segregation was demonstrated by the phenotype in the unaffected parents.

Targeted sequencing identified a novel *SCN2A* mutation in the patient. The mutation, c.4378G>C: p.G1460R (NM_021007.2), was in the coding region of exon 23 (Fig. 1). The mutation was absent in a human genetic variation database (the Japanese Genetic Variation Consortium: a reference database of genetic variations in the Japanese population, comprising 1208 individuals [http://www.genome.med.kyoto-u.ac.jp/SnpDB]), the 1000 Genomes project, National Heart, Lung, and Blood Institute (NHLBI) grant opportunity exome sequencing project (ESP), Exome Aggregation Consortium (ExAC), and in our 600 in-house control Japanese genomic samples. In silico analysis according to ANNOVAR, with predictions for c.4378G>C (p. G1460R), indicated a deleterious effect by SIFT (http://sift.jcvi.org/), Polyphen-2 (http://genetics.bwh.harvard.edu/pph2/), and MutationTaster (http://neurocore.charite.de/MutationTaster/). Sanger sequencing demonstrated that the mutation was de novo.

**Fig. 1:**
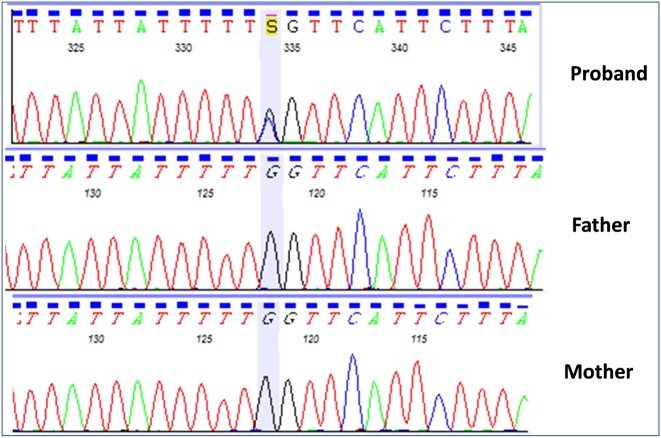
De novo heterozygous mutation in *SCN2A*, detected in our patient: c.4378G>C: p.G1460R

This patient showed a novel phenotype for heterozygous *SCN2A* mutations. *SCN2A* mutations are mainly responsible for various types of epilepsy syndrome, most of which are benign. Additionally, *SCN2A* mutations are rated second among gene mutations in nonsyndromic ID patients or ASD patients^6^. Both nonsyndromic ID and ASD are development-related diseases and sometimes overlap. Because there are few clinical descriptions of each case of these diseases, it is difficult to distinguish between them. A few cases with dysmorphic features and/or brain anomalies have been reported^7,8^. There were 221 pathogenic mutations registered as of April 2017 in the Human Genome Mutation Database (http://www.hgmd.cf.ac.uk), which were randomly distributed across the entire *SCN2A* gene. Of these mutations, most variants (181/221) were missense mutations. Missense mutations tend to lead to seizure development (Fig. 2a, b). The mutations causing nonsyndromic ID or ASD are variable (Fig. 2c). There is a tendency for nonsense mutations, splicing mutations, small insertions/deletions, and gross deletions to be responsible for nonsyndromic ID or ASD. However, regarding nonsyndromic ID or ASD and epilepsy, the genotype–phenotype correlation is still unclear. Although there are several cases of ID or ASD patients with heterozygous *SCN2A* mutations who also have seizures, little has been reported on the detailed clinical course^9,10^. At least, they have had seizures since the infantile period. The clinical course of our patient may represent a novel distinct phenotype of heterozygous *SCN2A* mutation syndrome. Further studies will identify the characteristic features of this phenotype.

**Fig. 2:**
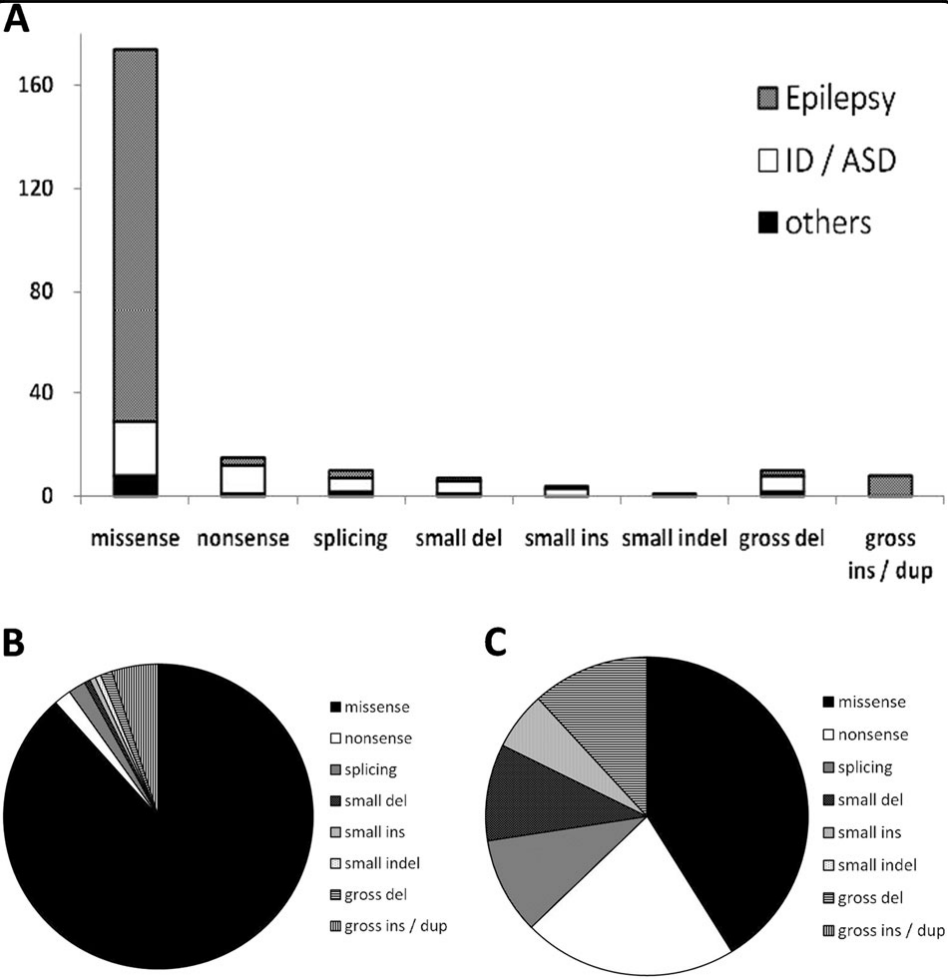
The clarification of mutations in *SCN2A*. **a** Correlation between *SCN2A* genotypes and phenotypes. The vertical axis indicates the number of mutations. **b** Mutations in *SCN2A* with epilepsy. **c** Mutations in *SCN2A* with nonsyndromic intellectual disability or autism spectrum disorders. del deletion, ins insertion, dup duplication, ID intellectual disability, ASD autism spectrum disorder

From a clinical standpoint, when managing nonepileptic ID or ASD patients with *SCN2A* mutations, clinicians should be aware of the possibility of seizure development. Genetic diagnosis may be useful to choose an antiepileptic medication. Furthermore, EEG must be performed regularly. As mentioned above, the genotype–phenotype correlation is not completely established. There are some reports that mention a correlation between mutated genes and antiepileptic drugs^3,11^. Further study can evaluate and predict the prognosis and the ratio of epilepsy in patients with *SCN2A* mutations.

In conclusion, we present a case of a nonsyndromic ID patient with a de novo heterozygous missense mutation in the *SCN2A* gene who developed seizures. This case suggests that nonsyndromic ID or ASD patients with *SCN2A* mutations are likely to develop seizures. This patient exhibited a novel phenotype for heterozygous *SCN2A* variants, and the case provides novel insights into the genotype–phenotype correlation for *SCN2A* variants. Further study is required.

## Data Availability

The relevant data from this Data Report are hosted at the Human Genome Variation Database at 10.6084/m9.figshare.hgv.2345.
